# A thematic analysis of adolescents’ perceptions of cyberbullying and cyberaggression: definitional distinctions and educational gaps

**DOI:** 10.3389/fpsyt.2026.1746672

**Published:** 2026-06-29

**Authors:** Emily J. Smith, Roshan Rai, Mark O. Scase, Amanda D. Wilson

**Affiliations:** Division of Psychology, Social Science Research and Innovation Institute, Faculty of Health and Life Sciences, De Montfort University, Leicester, United Kingdom

**Keywords:** adolescents, cyberaggression, cyberbullying, focus groups, social media

## Abstract

**Background:**

Adolescents are highly active online, which increases their exposure to harmful online behaviors, such as cyberbullying and cyberaggression. Extensive issues have been highlighted in the literature surrounding the conceptualization of cyberbullying, specifically those related to its definition, criteria and measurement. Cyberaggression was instead proposed to overcome these issues and adopt a more inclusive approach to researching online aggressive behaviors. However, despite the distinct differences between cyberbullying and cyberaggression, these terms are often used interchangeably leading to additional conceptual issues. This research explores how adolescents themselves perceive and understand the concepts of cyberbullying and cyberaggression.

**Methods:**

Three focus groups were conducted with 18 adolescents (aged 14-15) who used social media. Reflexive thematic analysis was used to analyze the data and explore common themes related to their perceptions of cyberbullying and cyber aggression.

**Results:**

Four main themes were identified: *Unclear definitional distinctions surrounding cyberbullying and cyberaggression*, *Unhelpful and unrelatable education*, *Complexities of the school environment*, and *Social media as a tool for perpetration*. The findings revealed that adolescents’ conceptualizations of cyberbullying were not consistent with academic definitions, and found it difficult to differentiate between cyberbullying and cyberaggression. Participants were dissatisfied with the outdated cyber-based education and identified barriers and concerns with reporting in their school. Adolescents’ perceptions were also largely underpinned by narratives of blame and responsibility.

**Conclusion:**

Practical implications concerning support and education are provided, though given the persistent conceptual issues with cyberbullying, adopting the broader construct of cyberaggression may provide a clearer framework for research, intervention and practice.

## 1 Introduction

Adolescents today are highly active online, with the majority using smartphones daily to access the internet for activities such as communicating with friends, listening to music, and engaging with social media (1). Although most social media (SM) platforms impose an age restriction of 13 years, SM use commonly begins around age 11, with children as you as 5 engaging with these platforms (2, 3). While online engagement offers many social and developmental benefits, it also increases exposure to harmful online behaviors, including cyberbullying and cyberaggression. In a report by The Children’s Society and YoungMinds (3), 9% of 11–12-year-olds, 21% of 13–15-year-olds, and 16% of 16–17-year-olds reported experiencing cyberbullying within a single month. Large-scale data indicates that cyberbullying victimization is associated with an increased risk of substance use, absence from school, and a range of mental health difficulties (4), underscoring the serious implications of online victimization. The conceptualization of cyberbullying emerged from the theoretical and definitional foundations of traditional bullying research (5). Understanding how these foundations translate into online contexts helps to provide critical insight into the conceptual evolution of cyberbullying.

### 1.1 From traditional bullying to cyberbullying

Bullying was conceptualized as a school-based aggression that became an area of research inquiry in 1970, defined as an aggressive behavior intended to cause harm, that is repeatedly carried out over a period of time, with an unequal power dynamic between the victim and perpetrator (6). As such, bullying is a viewed as a subset of aggression with the additional ‘special characteristics’ of repetition and an asymmetrical power dynamic (7). Whilst there is no standardized definition of bullying, the criteria of intention, repetition and power imbalance are widely accepted in the literature (8). The advent of the Internet and new communication technologies enabled bullying behaviors to occur in digital spaces, giving rise to what is commonly referred to as cyberbullying. Many researchers argue that cyberbullying is an extension of traditional bullying, due to an overlap in victimization (9–11), and similarities in predictors and outcomes associated with victimization and perpetration for both traditional and cyberbullying (12, 13). However, others argue that applying traditional bullying criteria to cyber contexts may be conceptually problematic due to the unique nature of computer-mediated communication (CMC) (14).

Cyberbullying is commonly defined as “An aggressive, intentional act carried out at a group or individual, using electronic forms of contact, repeatedly and over time, against a victim who cannot easily defend him or herself” (5, p. 376). This definition includes all traditional bullying criteria – aggression, intent, repetition and power imbalance – with the use of digital technology as the only exclusive characteristic (15). However, a review of adolescent cyberbullying research found that only 2 of 25 studies included all of these criteria in their definitions, highlighting the difficulty of consistently and comprehensively applying them across the cyberbullying literature (16). Technology can be used in many ways to perpetrate, initially via text messaging or emails, and more recently through social networking sites (17). However, scholars have challenged whether the traditional bullying criteria can be applied to the cyber context, as arguably the meaning of which do not translate well to online environments (8, 18–21). Variability and nuance in how the criteria are understood in online contexts is also evidenced in the literature. For example, repetition may occur through a single act of perpetration that is repeatedly shared (18, 22); intention can be difficult to determine due to limited social cues (23), but can be inferred from the persistence of the perpetrator or by adopting the perspective of a hypothetical, reasonable person (24); and power imbalance is understood as multidimensional, encompassing technological skills, target accessibility, anonymity, publicity and lack of control over dissemination (8, 15, 24–27). There are several other unique aspects of technology-facilitated communication that should be noted.

Differing from traditional bullying that typically occurs face-to-face, the social cues used to aid communication are absent from CMC (28), which can create a sense of deindividuation that leads to individuals engaging in less socially acceptable behaviors (e.g., cyberbullying) (29), make it difficult to perceive how communication has been received (e.g., whether harm has been caused) (30), and consequently reinforce these negative behaviors (31). The online environment also consists of unique factors suggested to contribute towards a disinhibiting effect that enables individuals to behave in a way, less governed by social constraints (32). Factors associated with the online disinhibition effect have been shown to be predictive of cyberbullying perpetration (33–36). Additionally, the ability to conceal one’s identity from the target (22, 27, 37–39), the relative lack of supervision (40) and publicity (15) further highlight the ways in which cyberbullying differs from traditional bullying through CMC. Targets are also far more accessible (22, 40), which due to the embedded nature of social relationships online, makes it difficult to escape/avoid being targeted without disengaging from SM entirely (41, 42).

### 1.2 Definitional inconsistencies and conceptual issues

Defining cyberbullying has proven challenging due to a lack of consensus regarding its definition and criteria, with researchers unable to agree upon a standardized definition (15, 18, 43, 44), reflecting its characterization in the literature as a complex and multifaceted phenomenon (45). Consequently, cyberbullying research is fraught with inconsistent findings that stem from the inconsistent conceptualization and operationalization of cyberbullying. Peter and Petermann (43) conducted a content analysis and found that 24 different cyberbullying definitions were developed and published between 2012 and 2017. However, only 50% of these studies provided information on how their definition was developed (e.g., based on previous literature, theoretical considerations etc.) and were not validated by further research. This definitional variability is further illustrated by a recent review identifying 43 author-defined conceptualizations of cyberbullying published between 2000 and 2024 (46). While electronic mediation, aggressive behavior, intention, and harm were more commonly included, repetition, power imbalance, and anonymity were far less consistently specified, highlighting ongoing conceptual ambiguity within the field. Furthermore, several reviews have identified substantial issues concerning variations in prevalence rates (44, 45, 47–50). In research published worldwide, cyberbullying perpetration ranged from 1.9% to 79.3% and victimization between 1.6% and 56.9% over a 6-month period (48). These variances were attributed to inconsistent definitions and conceptualizations, timeframes (e.g., past week, month etc.), and measurement tools (44, 47–49, 51). For instance, instruments used to measure cyberbullying often do not align with the conceptualization of cyberbullying and lack adequate reliability and validity testing (50, 51). All the aforementioned conceptual issues substantially impact the field, including clinical practice as UK clinicians require accurate prevalence’s rates to support adolescent mental health services associated with digital risks (52).

Efforts to establish a universal cyberbullying definition have largely relied on deductive, theory-driven approaches (18, 43), or on consensus-based approaches involving cyberbullying researchers (53). However, these approaches arguably overlook the important perspectives of those who engage in or witness behaviors associated with cyberbullying. As a result, researchers have advocated for these perspectives to be incorporated to ensure stronger conceptual alignment (14, 18, 43, 54, 55). Despite being the most researched group, there is limited research looking at how adolescents understand the concept of cyberbullying. Quantitative research indicates variability in criteria, as Menin et al. (55) surveyed Italian adolescents and found that dominance, frequency, and intention were key criteria for victims, while only dominance was identified for perpetration. Notably, omitting core bullying criteria. Spanish adolescents however, only consistently used power imbalance, intention and public dissemination to define cyberbullying, with differences again found between victim and perpetrator perspectives (54). Menesini et al. (56) asked adolescents to evaluate cyberbullying scenarios and found that intention and power imbalance (defined as the victim being upset and unable to defend themselves) were most important. However, their description of power imbalance may have influenced these findings by front loading victim impact. From a qualitative perspective, focus groups found that European adolescents defined cyberbullying based on intention to cause harm, repetition and whether the victim was affected (57). Similarly, participatory research with adolescents revealed frequency and intention were important criteria, though their interpretations were often ambiguous and subjective due to the online context (58). Using interviews, adolescent cyberbullying victims identified several defining features including victim impact, both deliberate and non-deliberate intent, repetition (whether multiple incidents or a single occurrence), use of technological medium, and publicity (59). Importantly, power imbalance was absent from these definitions, despite its prominence in traditional bullying definitions. Together these differing findings underscore the importance of recognizing how lived experience and prior victimization shapes adolescents’ conceptualizations of cyberbullying.

### 1.3 Cyberaggression as a broader concept

Based on many of the aforementioned issues concerning the cyberbullying definition combined with the vast array of aggressive behaviors that occur online, researchers have argued that a broader approach could be beneficial for research moving forward (14, 20, 60–63). Grigg (61) conducted focus groups and interviews with individuals aged between 8–54 to understand their perceptions of cyberbullying. Results suggested that cyberbullying was considered a vague, inadequate and restricted term, not broad enough to encompass the wide range of negative and aggressive behaviors that take place online. Instead, cyberaggression was proposed as a broader, more encompassing term, defined as “intentional harm delivered by the use of electronic means to a person or a group of people irrespective of their age, who perceive(s) such acts as offensive, derogatory, harmful or unwanted” (61, p. 152). This allows for a less restrictive approach to studying online aggressive behaviors, with cyberaggression acting as an umbrella term including behaviors such as: cyberharassment, cyberstalking, flaming and trolling etc.

Notably, cyberaggression moves away from the criteria repetition and power imbalance commonly associated with cyberbullying, making it a distinct construct. However, whilst researchers have started to adopt the concept of cyberaggression, it is being used inconsistently and interchangeably with cyberbullying (64–67). This is not a unique problem to just cyberaggression, as cyberbullying is commonly used as a more general term to also describe other cyber-based aggressions (e.g., cyberstalking and cyberharassment) (60). Along with the definitional distinctions, reporting differences have been observed when measuring cyberbullying and cyberaggression. Specifically, cyberaggression tends to show a higher prevalence than cyberbullying within the same samples (68, 69), indicating that these constructs represent related but distinct phenomena. Where intention, repetition or an imbalance of power are absent from cyberbullying research, such behaviors may be more appropriately classified as cyberaggression, given that these criteria are central to differentiate bullying from aggression more broadly (15, 24, 70). Additionally, there is also evidence of cyberbullying being used as an all-encompassing term (42, 44, 60). In response to this overgeneralisation, Olweus and Limber (71) stress the importance of not using cyberbullying as a blanket term to describe all forms of cyber-based aggressions.

Clarifying the conceptual distinctions between cyberaggression and cyberbullying would help to determine how these concepts are perceived by individuals outside the academic community, reinforcing the need to engage with target populations to ensure definitions align with the academic conceptualizations (14, 43). Despite qualitative approaches becoming increasingly relevant for understanding cyberaggression and cyberbullying, such research remains markedly under-represented in the literature (72). For example, only 7% of cyberbullying research published between 2000–2015 included qualitative data (73). Therefore, it would be beneficial to use focus groups (74) to understand how these concepts are socially perceived and constructed by adolescents. Thus, the exploratory research question in the current study is: how do adolescents perceive the concepts of cyberbullying and cyberaggression?

## 2 Materials and methods

### 2.1 Participants

A total of 18 adolescents (aged 14-15) participated in three semi-structured focus groups, which comprised of 16 females and 2 males with an average age of 14. **Supplementary Material Table S1** presents the participant demographics for each focus group. The SM accounts most commonly held by participants were WhatsApp and YouTube, followed by Instagram, Snapchat and TikTok. The lead researcher (EJS) worked with a gatekeeper at a non-selective foundation secondary school in the Midlands (UK) to recruit SM users aged 11-16. The school’s pupil population includes a larger proportion of families eligible for free school meals than the national average, which is commonly used as a contextual indicator of socioeconomic disadvantage in the UK education system (75). Given the exploratory nature of the research and the practical challenges recruiting adolescent participants, opportunity sampling was adopted. Participation required parental/guardian consent; therefore, the final sample comprised of the adolescents who were among the first to return their signed consent forms. The initial intention was to conduct focus groups across different year groups, whilst in keeping with recommendations for focus groups to maintain a 1–2 year age range between participants (74, 76). However, due to access issues within the school, focus groups were only conducted with 14–15 year old’s (Year 10). To maintain anonymity and external confidentiality (77) participants were assigned numerical codes (e.g., A01, A02) instead of pseudonyms. This approach was adopted to minimize the unconscious transference of characteristics that may be associated with names of a pseudonym (78).

### 2.2 Materials

The focus groups used a semi-structured interview schedule, developed to further explore the definitional and conceptual issues of cyberbullying outlined in the literature. Questions were formulated based on established methodological guidance (74, 76). They were reviewed by the authors, who have expertise in qualitative research and adolescent development, as well as an external researcher with subject expertise, to ensure appropriateness and consistency with the research aims. The final interview schedule comprised of 11 main questions, structured around three areas of focus to facilitate discussions on perceptions of cyberbullying, cyberaggression and distinctions between both concepts. A final question was also included to conclude the discussion and provide an opportunity for any remaining points to be captured.

### 2.3 Procedure

The school gatekeeper, who the first author (EJS) had a pre-existing professional relationship with, distributed parental information sheets and consent forms to interested students, to ensure the research team had no direct contact with prospective participants prior to parental/guardian consent being obtained. From the 47 parental/guardian consent forms distributed, only 18 were returned and participated in the focus groups. Following a returned consent form, the gatekeeper scheduled the focus groups and, on the day of each group, briefly introduced EJS to participants before leaving the room. All participants received an information sheet and completed an assent form. To keep the parents/guardians informed, the gatekeeper sent a text message notifying them of when the focus groups would take place. To build trust and rapport, EJS allocated time to engage in informal conversation with participants before starting data collection. The interview began with opening, introductory and transition questions about everyday technology use (74) to encourage early participation and establish a supportive, non-judgmental tone before moving to core topics of cyberbullying and cyberaggression. Focus groups were conducted in a quiet meeting room in the school between December 2019 and January 2020. The average duration of the focus groups was 64 minutes and 53 seconds, which included the ground rules and introduction. Focus group 1 lasted 52 minutes and 25 seconds, focus group 2 lasted 43 minutes and 35 seconds, and focus group 3 was the longest at 98 minutes and 40 seconds. Following each session, EJS wrote a brief personal reflection (79) in their reflexive journal, considering both the content of the discussion and their role as facilitator. Given the extended duration of the final session, the researcher also reflected specifically on factors contributing to its length. Several participants expressed that their participation was an enjoyable experience and appreciated the opportunity to have their voice heard, an observation documented in the reflexive journal and used to contextualize the analytic process. After evaluating the data quality, the richness of the data was deemed sufficient for addressing the research question so no additional focus groups were needed (80). All focus groups were audio recorded and transcribed to verbatim (81), using notations to record unclear and overlapping speech, laughter and pauses. Reflective notes were also made throughout transcription and each stage of analysis.

The research received ethical approval from De Montfort University’s Faculty Research Ethics Committee. As the research involved young people under the age of 16, ethical considerations in line with The British Psychological Society (BPS) Code of Human Research Ethics (82), Code of Ethics and Conduct (83) were adhered to throughout. This included informed consent from parents/guardians, written assent from participants that was continuously monitored, appropriate debriefs and clear safeguarding procedures should any disclosures be made during focus groups. As the researcher who conducted the focus groups would have direct, unsupervised access to the participants, a Disclosure and Barring Service check was obtained. The researcher who conducted the focus groups (EJS) clearly outlined the ground rules associated with maintaining confidentiality and being respectful of other participants views, prior to the focus groups beginning and were reminded of their right to withdraw at any point during the focus groups. No participants withdrew from the study.

### 2.4 Analytic strategy

Reflexive thematic analysis (84, 85) was use to analyze the data, as its flexibility supported the production of situated and contextualized knowledge by identifying patterns of meaning across the focus groups (86, 87). An inductive, semantic approach was adopted, consistent with the exploratory research question, whereby the analysis was driven by the data and themes represented participants’ explicit, surface level meaning. The analysis was underpinned by a relativist–constructionist orientation (88), which guided the exploration of participants’ perceptions of cyberbullying and cyberaggression as socially constructed realities shaped by their specific sociocultural contexts. This perspective emphasized the active role of the researcher in the production of knowledge, recognizing that meaning is co−constructed rather than discovered. Consistent with this analytical approach, reflexivity was embedded throughout all stages of the research process, with EJS using a reflexive journal to reflect on how her knowledge, disciplinary experience, and social and personal positioning shaped the research. EJS entered the study with the assumption that cyberbullying and cyberaggression are conceptually distinct yet overlapping concepts, but prioritized centering adolescents’ own interpretations when developing the themes.

The initial analysis was conducted by EJS, and themes were further refined and developed through reflexive discussions with the senior author (ADW). The different perspectives afforded by the varying levels of familiarity with the data and surrounding literature of both researchers, enabled a collaborative approach that added more depth to the constructed themes. The analysis followed the 6 stages of reflexive thematic analysis (84, 85, 87). The first phase required familiarization with the dataset, obtained through transcribing and thoroughly reading each transcript several times, making note of initial thoughts/observations in the reflexive journal. In the next phase, NVivo was used to code each data item based on key features that aligned with the research question. After the codes were collated, similar codes were grouped together to generate eight initial themes. These themes were further developed and reviewed, by continuously going back to the data to ensure they were consistent across all focus groups and addressed the research question. Of which four themes remained, with one theme comprising of two subthemes. The themes were given meaningful, descriptive names and were appropriately defined. Upon writing up the analytical narrative for each theme, the themes went through further refinement which led to the removal of the subthemes. Theme tables and coded extracts were reviewed by the research team to achieve investigator triangulation (89–91).

## 3 Results

**Figure 1:**
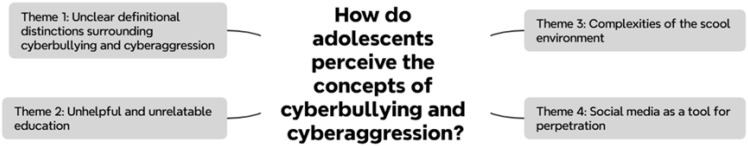
Theme visualization.

From the analysis, four themes were constructed to represent adolescents’ perceptions of cyberbullying and cyberaggression, across all three focus groups (please see **Figure 1**): *Unclear definitional distinctions surrounding cyberbullying and cyberaggression, Unhelpful and unrelatable education, Complexities of the school environment, and Social media as a tool for perpetration.* Themes presented below include a condensed supporting extract from each focus group. Please see **Supplementary Materials** for full extracts.

### 3.1 Theme 1: unclear definitional distinctions surrounding cyberbullying and cyberaggression

There was a range of variation surrounding definitional distinctions between cyberbullying and cyberaggression, with participants perspectives often being shaped by the consideration of different factors. This highlighted the difficulty of consistently differentiating between the two concepts. The participants found it noticeably more difficult to define cyberaggression in comparison to cyberbullying and often adopted slightly different perspectives depending on whether they were defining cyberbullying or cyberaggression. Cyberbullying was defined based on the perception of the victim and when it is affecting them:

A10: When you feel insecure about what they’ve said

A13: Yeah

A11: Or when it’s not just like (.) mentioned in a conversation where you’re having a laugh (.) where it’s just mentioned like you’ll just be talking about homework or something and they’ll just be like alright and then use a nickname that pokes fun at you

*Int: So a little bit unprovoked then?*

A11: Yeah

A10: Yeah

A13: Yeah

*Int: So just out of nowhere so there’s no kind of specific amount of times [no] you would say (.) do you just go by how you feel or?*

A11: Yeah

A12: Yeah it’s all personal as well because some people can go further and be able to deal with it more than other people

*Int: Okay*

A13: Yeah so it’s just whenever it hurts you

*Int: Yeah so then do you think then different people have different kind of*

A10: I think everyone has a different stopping point (.) so some person would just like say it once and be like please stop and someone could be like take it ten times and they want it to stop or they could just keep on taking it until they couldn’t take it anymore

When attempting to define cyberbullying and determine when an incident was classified as cyberbullying, there was a strong focus on the victim’s perception. Particularly in reference to the repetitive element. In response to the number of occurrences for an incident to be considered cyberbullying, this was based on whether the perpetrator continues “after you tell them to stop” (A09), or when “its clear you wanted them to stop” (A13), and was a shared sentiment by three other participants (A10, A11, A12). This could imply that occurrences prior to this do not count, as the perpetrator was unaware it was unwanted. It would also place the responsibility on the target to tell the perpetrator to stop or provide some clear indication of this, instead of focusing on the perpetrator who instigated unwanted behaviors. If and when the participants would do this was based on when they “feel insecure about what they’ve said”, it “hurts you” (A13) or if the insult was unprovoked and did not fit within the current conversation. There was no set number of occurrences as “it’s all personal as well because some people can go further and be able to deal with it more than other people” (A12) and “everyone has a different stopping point” (A10), thus making it difficult to have a standardized definition of cyberbullying. These perspectives offer an insight into how varied and subjective this threshold can be but also the emphasis on incidents needing to affect a person, to the point where they are unable to deal with it for them to confront the perpetrator and the incident be characterized as cyberbullying. Again, placing additional responsibility onto the victim.

When directly asked how they would define cyberaggression, this would often be met with difficulty:

A15: Making someone feel (.) unworthy

A16: Degrading someone

A15: Yeah

A14: Yeah

A15: Like (.) tryna make someone (.) oh its hard isn’t it when you actually think about it

A17: Belittling someone

A18: Yeah

*Int: Okay*

A16: Trying to make them feel bad

A15: Making someone feel small (.) just from words you say

The typical response when asked to define cyberaggression included long pauses, “ermm” (A15) or seeking further clarification which would suggest they do not know how to define cyberaggression, or that it requires more effort to form a response. This was further evidenced by verbally expressing how difficult they found constructing and communicating a definition, “oh its hard isn’t it when you actually think about it” (A15). Cyberaggression was then defined as “making someone feel unworthy” or “small” (A15), which highlights the negative impact on the target as a defining factor. Other descriptors included “degrading someone” (A15), “belittling someone” (A17) and “trying to make them feel bad” (A16), whereby other participants were also in agreement. All conceptualizations were centered around the perpetrator being the cause of the negative response to the behavior. The use of the words “make” and “making” also emphasized the active role and intention of the perpetrator in being the cause of the negative outcomes, rather than being defined based on when or if the target is negatively impacted. Which differed from the perspectives around defining cyberbullying evidenced in the previous extract.

Participants were later asked if they perceived there to be any differences between cyberbullying and cyberaggression, and suggested they overlapped:

A03: I think one leads to the other

A04: Yeah (.) it’s quite a fine line but (.) it yeah like online aggression just turns into online bullying eventually

*Int: Okay what do you girls think?*

A05: That (.) yeah like (.) either way online aggression you’re still criticising someone or something that they believe in or think that is right to them (.) and then you just carry on and carry on and then it just like becomes bullying and then like someone can’t take it anymore and they might just kill themselves

*Int: Yeah what do you think about it? Do you think they’re the same different?*

A02: It like depends on the level like (.) because aggression can be to a higher level or a lower level and bullying can be on a higher level and a lower level (.) but if they’re both on a higher level then it’s actually quite serious (.) if they’re both on a higher level

*Int: Do you think then they’re similar the same different or?*

A02: They’re quite similar because you’re still attacking someone (.) because of their thoughts or they’re views or because of the way they look

*Int: Okay yeah so is there anything else?*

A04: Probably just yeah (.) online aggression is just online bullying but (.) once online bullying is online aggression just several times

When deciding whether they thought cyberbullying and cyberaggression to be the same or different, an initial distinction made by participant A03 whereby “penalizing someone for themselves” would be indicative of cyberbullying, whereas targeting someone’s opinion was more related to cyberaggression. However, this distinction reflects victim blaming as the participant implied that cyberbullying occurs as a punishment or consequence of who the individual is, while cyberaggression arises as a consequence of someone’s opinion. Nevertheless, cyberbullying and cyberaggression were thought to “overlap” as “one leads to the other” and would suggest that the two concepts are not entirely distinct. A view that was also supported by participant A04 who described the distinction as a “fine line” as “online aggression just turns into online bullying eventually”. This indicates a perceived progression from cyberaggression to cyberbullying, though how that line is crossed was ambiguous and could be influenced by their ambiguous cyberbullying definitions and difficulty conceptualizing cyberaggression. When focusing on the perpetrators perspective, they were still “criticizing someone or something” (A05) and thus the behavioral actions and intention between cyberbullying and cyberaggression would be similar. The behavior then only crosses the line and “becomes (cyber)bullying” (A05) when the perpetrator “carry on and carry on” (A05). At this stage, the target “can’t take anymore and they might just kill themselves” (A05). This frames the progression from cyberaggression into cyberbullying as a result of the targets inability to endure the aggression, rather than the perpetrators actions, again placing additional responsibility onto the target. The phrasing “they might just kill themselves” further highlights this as it positions the act as something the target does to themselves, rather than a consequence of the perpetrator’s actions. Distinguishing between cyberbullying and cyberaggression became more difficult when seriousness or “level” was used as the criterion. Participant A02 described how both “can be on a higher level and a lower level” and that only “high level” incidents were “quite serious” and a cause for concern, whereas “lower level” incidents were dismissed. Although, as both involved the perpetrator “attacking someone” (A02), cyberbullying and cyberaggression “were quite similar”. This distinction reflects a focus on the nature or intention of the act, rather than the frequency. In contrast, participant A02 further elaborated on their earlier point that there is a “fine line” and centered the distinction around by the frequency, noting that “online aggression is just online bullying but once, online bullying is online aggression just several times”. This highlights how distinctions between cyberbullying and cyberaggression can be shaped by multiple factors, including the nature of the act, the perpetrators intention, severity and frequency.

### 3.2 Theme 2: unhelpful and unrelatable education

Within the focus groups, participants shared their thoughts on the cyber-based educational resources within their school environment. These were typically described as outdated, unrelatable and unhelpful. Due to constantly evolving technologies and features on SM, the educational resources were not able to keep up and be representative of current online behaviors experienced/witnessed by adolescents. The participants reflected on the online safety education within their school:

A10: They’re all outdated

A11: Yeah

A13: Yeah

A10: They’re like (.) Blackberry’s from like 2007 [yeah] they should at least use an iPhone or like anything newer (.) to make it show how you can do it that phone instead of a little Nokia brick

A11: I swear on one of them they’re on flip phones you know

A12: Yeah

A10: Exactly

A09: Yeah like the only updated one was that one was that girl on the MacBook thing (.) on the laptop

A12: Yeah but that one was quite old though still

A09: Yeah but still

A11: I think the most recent one we’ve had is when the police came in and they were like (.) oh whatever her name was and this man they were texting and then he chased her through the forest

A10: Yeah that was the newest

A11: I think that was the newest and that still happened what 2015 ish or something like that

The participants provided several examples of the online safety videos they were shown within their school. Of which the behavioral scenarios presented in the videos were behaviors the participants have “never seen happen” (A11) and they could not consider a time when “that ever happened” (A12), building an argument around how relatable these behaviors were to the participants. They also described the behaviors as “really weird” (A12) and that they “don’t make sense” (A09), evidencing how critical the participants were of these educational videos. When trying to provide an example specific to cyberbullying, a participant said “they never actually do cyberbullying” (A12) and instead they “they always like promote the dodgy men ones” (A11) which provides an important insight on the types of videos shown within the school. Thus, would suggest that more commonly experienced behaviors may not be part of the online safety education within the school, due to the videos being “all outdated” (A10). In addition to the behavioral scenarios being “outdated”, the technology used within the videos was also outdated and demonstrates another angle whereby the educational resources were unrelatable. The participants repeatedly emphasized this by giving examples the outdated technology: e.g., “Blackberry’s from like 2007”, “a little Nokia brick”, “flip phones” and stated they could “at least use an iPhone or like anything newer”, as this would make the video more relatable and as a result, useful. The video that used a “MacBook thing”, was the most up to date technology, however that was “still quite old though”. There is a clear issue around the need to more updated educational videos, that present relatable scenarios using relatable technology.

Participants also discussed not knowing who their student council members were or the role of this group in combatting bullying (both traditional and cyber), along with the usefulness of anti-bullying posters throughout the school:

A15: They get told to (.) like when I was in school council it was 5 minutes just talking about bullying but what do they actually do

A14: They just use the kids

A15: They can have a poster erm (.) if I’m gay I’m gay for homophobic (.) if I’m this colour I’m that colour if I’m this religion

A17: If I’m this colour I’m that colour (laughs)

A15: It’s true but (.) I could walk around the school and read the posters (.) but what’s the posters gonna do to me (.) make me read it (.) is it gonna make other people stop no

A16: Improve your English skills (laughs) () It’s like they don’t think about the actual

A15: Yeah (.) I sometimes think that a group of people should come together and do an assembly for each year to show how it can affect people and say their own

A17: Yeah (.) we need actual people to come in school

A16: It needs to be personal so that it gets to us

A17: It can’t just be a teacher that’s like I went to school years ago it was easier its hard now (.) deal with it (.) it’s like

Despite there being student council members, whose role involved being a point of contact for students to flag issues to bring to dedicated meetings, four participants (A14, A18, A17 and A15) expressed not knowing what the council members actually do, and one student (A18) did not know who the council members were. There was also no mention of the student council in FG1 or FG2 which could imply that they too are unaware of their role in relation to flagging concerns regarding cyberbullying or cyberaggression. In participant A17’s previous role as a council member, they stated that “it was 5 minutes just talking about bullying but what do they actually do” implying that even they were not convinced of their role in relation to bulling (traditional and cyber). Continuing with the unfavorable narrative of the student council, another student believed “they just use the kids” (A14), suggesting the student council was not of a benefit to the students within the school. The school had educational posters displayed throughout, aimed to educate students about homophobia and religion which was an example provided by A15. However, this led to them thinking “but what’s the posters gonna do to me … Is it gonna make people stop no”. Which provides an insight into how useful they believe the posters are in preventing bullying. The participants then proceeded to made a joke around the posters only being useful for improving reading comprehension skills for those who bother to read them. Based on being dissatisfied with the role of the student council and the posters around their school, the participants wanted to be proactive and have an input in how anti-bullying initiatives were delivered and by who to ensure their needs were being met in a relatable, meaningful way. They believed having “a group of people should come together and do an assembly for each year to show how it can affect people” (A15) and having “actual people to come in school” (A17) would be a better approach to combatting cyberbullying. They also believe that education “needs to be personal” (A16) and should not be delivered by a teacher. As having a teacher who does not understand the potential effects of cyberbullying and advise the students to “deal with it” (A17), which was unhelpful and minimized the feelings of the students.

Despite being dissatisfied with the online safety education within their school, the participants still believed that education was an important and necessary resource to reduce prevalence rates and encourager safer SM use:

A03: I don’t think you can prevent it to be honest

A04: Yeah yeah you can’t [okay] cause like think how many millions of people are using social media and things like that (.) if one of your tweets or anything or one of your posts blows up (.) because you’ve said something (.) then people have that people will save that people will screenshotted that (.) you’ve gotta (.) you know take it or just don’t say anything like that

A03: Like I think the best shot is to educate people more (.) but even then (.) you know you can minimise it if everyone stops using social media which is not gonna happen (0.2) it’s the same with limiting screen time (.) you can’t really do anything to do that (.) cause like kids are gonna use social media regardless of what you put in place so I guess that’s the same with like aggression maybe

A04: And the behaviours becoming like more acceptable with like young kids because you see like a lot of (.) you know 10-year olds with phones with like all these different accounts (.) they’re now exposed to all those kind of behaviours that adults are displaying (.) and they think that’s acceptable

Whilst discussing cyberbullying prevention, participants were generally skeptical regarding whether cyberbullying could be fully prevented. Participant A03 explicitly stated “I don’t think you can prevent it to be honest”, and the use of the word “you” could imply a perceived personal responsibility in trying to prevent such behaviors. This was echoed by participant A04 who agreed that “you can’t”, which could again further reinforce the notion of individual responsibility and accountability. This participant further elaborated on how posts can “blow up” beyond your control and receive a lot of attention. In these instances, individuals should tolerate any consequences of this additional attention and just “take it” (A04), or refrain from making such posts and “just don’t say anything like that” (A04), which further reinforces the idea that it is the individual user’s responsibility to avoid being targeted. It could therefore be argued that receiving appropriate education or guidance that goes beyond self-regulation practices, may help challenge the belief that they simply have to “take it” (A04). The need for more comprehensive education was supported, as participant A03 believed “the best shot is education” as this may help to “minimise” cyberbullying incidents. Though, they did acknowledge that total prevention could only be achieved “if everyone stops using social media”, a solution that is not realistic. Upon reflecting on advice around “limiting screen time” (A03), this was also considered impractical as “you can’t really do that”. Thus, suggesting that cyberbullying education should instead present realistic and practical solutions or advice. The need for improved education was further underscored by concerns about the early exposure of young users to aggressive behaviors online. Participant A04 highlighted how normalized and common such behaviors are, noting that even “10 year olds” are being “exposed to all those kind of behaviors that adults are displaying” and as a consequence will learn from these patterns of irresponsible SM use exhibited by adults. This further reiterates the need for education to promote appropriate SM use, and challenge the belief that individuals are primarily responsible for preventing cyberbullying. Although the participants frequently critiqued the current online safety education within their school, it is encouraging that they still believe in the potential value of more effective, relatable education.

### 3.3 Theme 3: complexities of the school environment

As secondary school students, the participants reported how when an individual experiences an act of cyberbullying or cyberaggression, careful consideration must be given to how they respond and the reporting method chosen. Each method for reporting carried distinct implications and potential repercussions within the school environment, which in turn, would influence their reporting actions:

A17: Yeah but then they complain (.) they complaint about the negative things they’ve got like (.) right either delete the negative things (.) or don’t answer it (.) or don’t do the ask me a question thing again

A14: Or block them (.) like so many people keep them people on their Instagram’s and their Snapchat’s because they want an argument (.) it’s not the fact that [yeah that’s true] they could be crying to their friends (.) they could be crying to their mums and dad but yet (.) they’re still on your Instagram able to send these comments able to look at your (.) stories and your photos so what’s the point (.) block them

A17: Yeah but also at the same time (.) like sometimes blocking them or unfriending them causes a bigger argument

A15: Yeah it makes no sense because like

A17: Especially if you’re in school (.) if you like (.) say you block someone like (.) out of school (.) they’re gonna come up to you and ask you why you’ve blocked them (.) like its (.) sometimes it’s not worth it

A15: Or they’ll tell all your friends and then they’ll ask you

A17: Yeah like can you ask so and so like no I did it for a reason

A common recommended response to cyberbullying or cyberaggression is often to report, block or delete the perpetrator. This was stated in discussions across all three focus groups for a range of behaviors. Following a discussion about use of the “ask me a question thing” (A17) on Instagram and Snapchat, there was a sense of judgement towards those who “complain” about receiving negative comments, as participant A17 believed they should simply ignore or delete the comment, or refrain from using that function. Which alludes to a sense of victim blaming and acceptability of a negative comment being a normal outcome of using this function. Alternatively, participant A14 suggested to block the perpetrator. Individuals who did not follow this course of action were considered to “want an argument”, even if they are “crying to their friends” or “crying to their mums and dads” (A14). This demonstrates how targets who do not act in this way when victimized are judged by others for their lack of action, and are again placed with the responsibility of preventing further incidents by adjusting their SM use. However, blocking can lead to further issues and “causes a bigger argument” (A17). Especially with the addition of the school environment as “they’re gonna come up to you and ask you why you’ve blocked them” (A17), and the use of “gonna” could imply this is believed to be a likely outcome. There was agreement from participant A15 who said “it makes no sense” to block them, despite earlier in the conversation saying “delete them (.) block them (.) gone”, which could insinuate that the problem is “gone” once you have blocked and deleted the perpetrator. Friends of the perpetrator may also get involved, thus the likelihood of being approached within the school environment could lead to additional distress for the target. Due to these added complexities of the school environment when blocking or unfriending perpetrators, this action was viewed as “not worth it” (A17).

When reporting to a staff member at the school, the participants would carefully consider who they report to, in attempt to be heard and face minimal potential repercussions:

A06: Tell the teachers (.) there’s more teachers than students

A04: There you go you don’t fight

A05: If you go to the teachers they’re gonna get other people and then it will escalate even more

*Int: So you don’t necessarily think telling the teachers is a good idea?*

A05: No because they don’t do anything

A01: They don’t really do anything

A05: Not most of them do anything but it will just escalate even more because they’ll be like because you’ve told the teacher I’m gonna get told of now and then they’ll just do it even more

A02: It depends what level they are like (.) head teacher teacher or like support

A06: Yeah but if you tell the teacher then they’ll probably escalate it to out of school (.) and then like get you when you’re out of school

A03: But even if you do tell a teacher they can’t physically from that voicing their opinion (.) so it’s more you just need to be sensible and like back down

A02: You’re not gonna back down if you’re right are you?

A03: Yeah but then it’s sensible if they won’t shut up

A04: You just have to be mature yeah

A03: Yeah there’s no point just getting your hands dirty (.) just to fall to a lower level

A02: It depends how much it annoys you (.) if you like just think (.) its constant and you now it’s not true (.) not speaking the truth and then you can just leave it

Similarly to blocking a perpetrator on SM, participant A04 felt that reporting to a teacher would cause the incident to “escalate even more” as the perpetrators are “gonna get other people” involved. A point they later reemphasized. Participant A06 shared this perception and stated that the perpetrators would “escalate it to out of school” and “get you when you’re out of school”, indicating the potential for reporting to a teacher leading to physical aggression. As teachers also cannot “physically” stop people from voicing their “opinions”, participant A03 and A02 thought it was more “sensible” to “back down” and “be mature”. This insinuates that reporting to a teacher is immature, and places a burden on the victim to respond to the incident in a ‘mature’ manner. There were discussions across all focus groups around the effectiveness of this action, as participants A05 and A01 said the teachers “don’t really do anything”. Although, participant A02 suggested that the response from the teacher could be determined by “what level they are”, e.g., “headteacher or classroom support”. When later asked to elaborate on these levels, participant A04 thought they would feel more comfortable speaking to a teaching assistant than an “actual teacher” as they would be “more understanding”. There were teachers that were thought to “listen” and others that would “be like oh well” (A06). Other participants said they would go to the teachers that they believed to like them as well as the teachers that were “nice” (A05), as “some teachers don’t like you” (A01), which was a sentiment shared across all focus groups. The response to the incident was also guided by “how much it annoys you” (A02) to perhaps consider whether the implications of reporting to a teacher were worth it. This would imply that when deciding whether or not to report an incident of cyberbullying or cyberaggression, careful consideration needs to be made as reporting could cause the situation to escalate, in addition to appropriately choosing who to report the incident to.

More serious incidents reported to the school had previously resulted in police involvement, an outcome viewed as unhelpful and as a result, served as an additional consideration when deciding whether to report an incident:

A11: On social media but if you go to a teacher in school and be like oh so and so say this online or blah blah blah is doing this (.) most of the time (.) instead of like sometimes in really serious cases they call the other person up but a lot of the time they’re just like block them or report them [yeah] so it’s just not helpful it’s just like

A09: Or they get the police in school and you have a meeting (.) that’s what happens to most people

A13: Yeah depending on how serious it is

A09: Yeah

*Int: Oh so if it gets quite bad they get the police in to talk to [yeah] the person who’s doing it?*

A09: To both of them

A10: To both of you together in the same room

*Int: Oh okay (.) do you think that’s useful or?*

A10: No

A09: No

A11: No

A12: I have never been involved in it to be fair

Consistent with previous discussions, typical responses from teachers when reporting resulted in the recommendation to block or report the perpetrator which was not a “helpful” response. However, in more serious cases, participant A09 explained how the teachers would “get the police in school and you have a meeting”. A seemingly common outcome that “happens to most people” (A09), but depends on “how serious it is” (A13). In such cases, the police would come into the school and bring those involved together in the same room to discuss the incident. The participants were in agreement that this was not a useful strategy. When later asked if they would feel comfortable reporting an incident knowing the police may get involved participant A11 said “no not really” as it would be “stressful for those people”. Whilst the incident could be serious, they questioned whether it was “have to get the authorities in bad” as the individual who reported the incident might think “I’ve let this happen” and feel guilty for the police being involved as a consequence of that action. The unfavourable views towards police involvement in facilitating a discussion between those involved was not only another factor that could deter them from reporting, but also added to the complexities of dealing with incidents of cyberbullying or cyberaggression in the school environment.

### 3.4 Theme 4: social media as a tool for perpetration

Individuals who perpetrate cyberbullying and cyberaggression were suggested to utilize the functions and accessibility afforded by SM to aid perpetration. This not only makes it more difficult for targets to manage or escape the incident, but it also allows perpetrators to avoid facing consequences for their actions. The participants explained how the anonymous messaging feature on Snapchat was commonly used to send aggressive messages:

A12: There are a lot of negative things like especially (.) on peoples (.) anonymous things you get a lot of (.) bullying for like LGBT reasons

A11: Yeah and there’s just like (.) a lot of people just because they’re anonymous saying a lot of things

A12: Because they don’t have the face to actually say it

A11: Yeah

*Int: Oh okay so this is on snapchat?*

A12: Yeah

*Int: And you don’t necessarily know who the person is?*

A10: No cause you can do this thing and then you can send an anonymous message

*Int: Oh can you?*

A11: Yeah a lot of people send like nice things

A09: Yeah you put like ask questions

A10: And then they just send stuff

A09: Yeah or they just put like negative comments

A12: Yeah some of them are nice and then obviously some just go a bit far

*Int: Is that a little bit like the thing on Instagram where you ask the questions*

A09: Yeah

A10: Yeah

A12: Yeah

A11: Yeah

A10: It’s like that but you just can’t see the people who it is

When reflecting on the types of aggressive behaviors observed online, the participants referred to anonymous messages on Snapchat. Participant A14 mentioned how such features often contain “a lot of negative things”, particularly towards LGBT individuals suggesting that these anonymous features facilitate homophobic messages. Building on this, participant A11 shared how “a lot of people” say “a lot of things”, with the repetition of “a lot” emphasizing how often this feature is used for sending aggressive messages. Anonymity was thought to allow individuals to say things “they don’t have the face to actually say” (A11) and provides an opportunity to send an aggressive message that may not have otherwise been sent. In contrast, the anonymous message feature was not seen as inherently negative or aggressive, as the participants did acknowledge that “a lot of people send like nice things” (A11) but then “some just go a bit far” (A12). Participants explained that a similar feature exists on Instagram, however, users were not anonymous. Notably the participants did not reference this when discussing examples of aggressive online behaviors, suggesting that the lack of anonymity may limit the use of this feature for sending negative messages. Further discussions evidence how receiving anonymous messages can be “overwhelming” (A13) and prompt feeling “really insecure” (A11) due to being unable rationalize why the message was sent (e.g. “they don’t like me”). This demonstrates how this particular Snapchat feature was utilized by perpetrators and empower them to send an aggressive message whilst concealing their identity and protecting them from being faced with consequences.

Private stories were another example of SM features used to facilitate aggression:

A17: And private stories (0.2) private stories have a lot of gossip

A15: Private stories are the worst because you put people on your private (.) that they shouldn’t be the people on the private story because they’ll then go and tell the person (.) like

*Int: So is that like the close friend’s bit where you can just send it to certain people?*

A17: Yeah

A15: Yeah it’s on Snapchat as well but you can have [oh okay] like as

A17: Snapchat you can have like loads

A15: Close friends you can have like thirty but Snapchat you have like (.) 300 or as many as you want to be honest

*Int: Oh okay (.) so people send like private gossip or something?*

A14: Yeah

A15: Sometimes or they’ll just put like where they are and what they’re doing but they don’t [yeah] they don’t want all their followers to see (.) just certain people

A17: Yeah

Building on the previous example, participants also highlighted the use of private stories as a medium for cyberaggression. Prior to this, they inferred that Snapchat had “been made to send nude photos”, but failed to acknowledge how sharing or re-distributing nude images as an adolescent was a criminal offense, highlighting another potential shortcoming for cyber-based education in the school. Most SM platforms now allow stories to be shared with a select amount of people, specifically chosen by the user and frequently has “a lot of gossip” (A17). Participant A15 described private stories as “the worst” (A15) as typically, the people the gossip is about will be excluded from the story, though will occasionally include users who will “go and tell the person”. Which could imply that the issue with doing this may not be related to the indirect act of aggression itself, but it getting back to the target. Snapchat appears to be about to facilitate this on a higher level due to allowing “300 or as many as you want” (A15) in comparison to the perceived limit on Instagram’s close friends. This illustrates how private story functions can facilitate exclusionary behaviors.

Following an aggressive incident, participants explained how blocking or reporting the perpetrator is not always effective:

A02: Probably report it and then block them (.) keep the evidence

*Int: Okay so how would you report it?*

A02: Probably at the report button

*Int: Oh so like on the social media account you’d report it that way*

A06: Sometimes that doesn’t work though

A01: Block um

*Int: The reporting?*

A01: Yeah because they make new accounts and then harass you

A06: And put your account on private

A04: I feel like (0.3) a lot of people (.) it’s not very like proactive (.) a lot of people say (.) I am gonna block them and keep the evidence and report it or whatever (.) and it just doesn’t happen (.) I don’t think I’ve ever seen someone actually like report someone because they’re being attacked or whatever

A06: My cousin does it all the time

A04: On social media

Blocking or reporting the perpetrator via the SM platform, while also retaining evidence was a common response when asked how the participants would respond to being a target of these aggressive behaviors. Though participant A02 suggested that this “doesn’t work though”, with participant A01 adding that perpetrators “make new accounts and then harass you”, making blocking and reporting ineffective and useless. Making your SM account “private” (A06) was another option, though this did not seem to be a shared sentiment due to the lack of agreement from other participants and mentions across the other focus groups. Reporting was also perceived as uncommon as participant A04 said they “don’t think I’ve ever seen someone actually report someone”. Although this is a private process so would not be typically seen by someone outside of those involved, unless shared as with participant A06’s cousin who “does it all the time”. Thus, suggesting variation in the usage of this feature. Nevertheless, reporting perpetrators could arguably be seen as ineffective and consequently individuals become less “proactive” about reporting due to perpetrators ability of bypassing any restrictions intended to limit their access to the target. Overall, this would indicate that perpetrators are afforded a new type of power by SM platforms which afford them unrestricted access to their target and reduce the effectiveness of protective measures.

## 4 Discussion

To address the research question, the study provided an in-depth exploration of how adolescents perceive the concepts of cyberbullying and cyberaggression in interactive focus groups. This was the first time that qualitative focus group research has investigated adolescent’ conceptualization of these concepts together. When attempting to define the concepts, adolescents’ perceptions were ambiguous, either adopted the perspective of the perpetrator or the victim, and did not always refer to the core bullying criteria. As a result, distinguishing between acts of cyberbullying and cyberaggression was somewhat challenging as the concepts were seen to overlap. All discussions were centered around SM and how perpetrators utilize different functionalities to facilitate aggression, have unrestricted access to their target and avoid repercussions for their actions. The findings further suggested that the education which covered cyber-based aggressions was outdated and unhelpful. Therefore, they could benefit from education that is more relatable – from a technology, represented behaviors and delivery perspective – as this was key to developing awareness, prevention efforts and support for those targeted. Whilst they were able to identify the commonly advised strategies for prevention and actions following an incident (e.g., block and report perpetrators), more attention was needed around the additional difficulties associated with dealing with cyberbullying or cyberaggression within the school environment. The themes will be discussed in relation to pre-existing literature, with a particular focus on the strong undercurrent of victim blaming and responsibility; practical applications will be provided, followed by a consideration of the study’s limitations and suggestions for future research.

### 4.1 Conceptualizing cyberbullying and cyberaggression

In relation to the conceptualization of cyberbullying and cyberaggression, the participants perceptions of these concepts were not entirely consistent with previous literature. When discussing definitions of cyberbullying, this was heavily focused on the target’s perspective. For example, if they were affected (41, 57, 59) and determined intention based on whether the perpetrator continued after being told to stop (8), with very little mention of the perpetrator’s actions. This consequently led to subjective perceptions due to differences in people’s tolerance of handling these aggressive behaviors. There was little mention of power imbalance or repetition in discussions around defining cyberbullying, despite these being defining features for adolescents in previous research (54–57, 59). In contrast, the disregard of power imbalance is congruent with previous research (55, 59). By adopting the perspective of the victim, the adolescent’s views align with Alipan et al. (18) multiple perspective approach, where a cyberbullying definition was proposed which encompasses the perceptions of the perpetrator, victim, and bystander. Within this definition, the victim perspective emphasizes intentional harm as a defining feature, consistent with adolescents’ perceptions in the present study, and suggesting they may already adopt this definitional approach.

With cyberaggression, participants found this more challenging to define, often showing uncertainty on what cyberaggression actually is. However, unlike cyberbullying, participants typically focused on the perpetrator (as opposed to the target) and their intention to cause harm. As such, their perceptions closely aligned to Grigg’s (61) definition of cyberaggression. When attempting to distinguish between cyberbullying and cyberaggression, participants did use some of the bullying criteria – such as intention and frequency/repetition – in addition to the nature of the act and severity to differentiate. Though they believed the concepts overlap and there was a fine line between the two. This research provides evidence that the concepts are also conceptually confused amongst adolescents (64–67). As the participants were transparent about their views of the school’s cyber-based education and provided examples to illustrate how outdated and unrelatable they felt it was, it is reasonable to assume these perceptions were their own and not heavily influenced by any education, interventions or awareness campaigns in the school. Based on the conceptual issues and inconsistencies highlighted in previous literature and how the adolescents’ perceptions of cyberbullying were missing the core bullying criteria – argued to be important distinguishing factors for differentiating from cyberbullying and aggression (15, 24, 70, 71) – cyberaggression could be a better concept to adopt in research moving forward.

### 4.2 Education and support

The participants were not satisfied with the current cyber-based education within the school and stressed the need for educational resources to be relatable and in-keeping with current technology. Whilst it is important to acknowledge the difficulty of educational resources keeping up with the constant advances in technology and SM platforms, this was consistently emphasized by the participants. Looking at anti-bullying educational programs more generally, a meta-analysis concluded that when programs included activities that involved peers (e.g., classroom discussions), these were more effective at reducing bullying perpetration and victimization (92). Whilst this focuses on bullying interventions more generally, some of the included interventions did cover cyberbullying so could be relevant to apply to cyber-based educational programs. Especially given the adolescents strongly endorsed the need for relatable education, which could be achieved by utilizing peer involvement. Participants referred to posters used in awareness campaigns and phrases such as “stop bullying” which were not well received. In contrast, Spears et al. (93) codesigned cyberbullying awareness campaigns with young people, to successfully enhance the relevance, authenticity and impact of the campaign messages. This further highlights the importance of involving young people to achieve desired outcomes for cyberbullying initiatives in schools.

The school environment also posed additional challenges when experiencing cyberbullying/cyberaggression victimization, due to the risk of physical escalation at school, challenges in carefully deciding if or whom to report incidents to, and concerns about the consequences of reporting. Consistent with previous research, many young people refrain from reporting cyberbullying victimization, due to fear of retaliation, escalation, or the loss of digital connection (94, 95). Notably, reporting rates to school staff remain low, with only 7.56% of UK adolescents indicating they would report to school personnel (69). Participants in this study had minimal faith in teachers’ ability to effectively manage cyberbullying, often choosing to approach only staff members who they perceived as nice or understanding, thus underlining the importance of approachable and informed school personnel who are aware of their responsibility to manage reports in a supportive, effective manner.

This lack of student confidence mirrors research with teachers themselves, who report insufficient knowledge and confidence in dealing with cyberbullying incidents (96). Evidence from a content-analysis of 200 anti-bullying policies in UK schools further reinforce these concerns, and revealed policy coverage should be improved to better highlight the responsibilities of other school staff and include guidance on dealing with cyberbullying specifically (97). Comprehensive anti-bullying policies are legally mandated in UK schools (98) and best practices encourage the involvement of educators and parents when writing policies (99). Though this neglects to consider the inclusion of student voices. To the authors’ knowledge, there is no empirical evidence supporting student involvement in co-producing cyberbullying policies or initiatives in schools. Therefore, this study provides novel evidence to support greater student involvement in policy development, or at a minimum, be given opportunities to provide feedback on existing policies. Though research is limited, there are examples of existing initiatives within third sector organizations that advocate for the inclusion of the student voice in developing/reviewing anti-bullying policies and resources include free to access materials produced by The Diana Award (100) and The Anti-Bullying Alliance (101).

Beyond the school context, research has noted many negative psychological consequences associated with online victimization including post-traumatic stress (102), diminished self-esteem (103), and mental health outcomes such as anxiety, depression, suicide ideation, self-harm (104). As such, clinical intervention following victimization may be required. Yet clinicians in the UK expressed challenges around knowing how to encourage incident reporting and felt unable to appropriately advise to young people (52). Similar knowledge gaps were found with therapeutic practitioners (105). To maximize the effectiveness of interventions, having young people involved in educating clinicians about online harms was emphasized (52). Overall, there is a strong rationale for utilizing input from adolescents to strengthen education and support in both schools and clinical or therapeutic practice.

### 4.3 The role of social media

All focus groups centered discussions of cyberbullying and cyberaggression within the context of SM, echoing previous research stating that SM provides ample opportunities for cyber-based aggressions (59) and empowers perpetrators (94). Functionalities across different SM platforms were suggested to aid perpetration, particularly with the anonymous messaging feature on Snapchat. Whilst a similar feature without anonymity does exist on Instagram, cyberaggression was not as commonly reported in this sample. Thus, anonymity feeds into the disinhibitory effect (32) and consistent with previous research is linked to increased cyberbullying perpetration (22, 33–36, 38, 106). Therefore, such features are enabling and encouraging undesirable use. The perceived inability to escape cyberbullying or cyberaggression on SM was a significant concern among participants, who inferred that perpetrators could circumvent protective measures like blocking or reporting by creating new accounts or switching to different platforms to gain access to their targets. This skepticism extended to doubts about the effectiveness of the reporting functions themselves, as many were not convinced that this would lead to consequences for the perpetrators, thereby rendering these protective mechanisms ineffective. Literature supports these barriers to reporting and has identified several factors that influence an individual’s likeliness to use these functions, including whether they perceive the reporting function as useful, feel a personal responsibility to take action, their personal capacity to tolerate the abuse has been surpassed, hold positive attitudes towards the reporting function, believe the content requires urgent intervention, and feel they have control over the reporting action (107–109). These findings illustrate the complex nature of reporting behavior and how there are many reasons why individuals may not utilize these functions. Moreover, there are ongoing critiques of SM companies due to their inconsistent and inadequate response to cyberbullying, and their lack of transparency in how reports are moderated (3). Thus, SM companies should also consider the negative implications of its features and ensure that appropriate actions are employed following reports.

Participants also highlighted how normal it was to be exposed to cyberaggression on SM. Particularly, how they had observed adults also using SM irresponsibly and believed this contributed towards young people learning that this was a normal and acceptable way to behave on SM, and so mirror those behaviors themselves. When the focus groups were conducted, President Donald Trump was frequently seen engaging in risky, aggressive behaviors on Twitter (110) without consequence, and was repeatedly bought up by the participants. Previous research with adolescents indicates that social norms can predict intention and engagement in cyberbullying perpetration (111, 112). Furthermore, individuals who hold stronger views around the acceptability of cyberaggression were also more likely to engage in cyberaggression perpetration (113–115). These findings further highlight how the normalization of cyberaggression and cyberbullying on SM needs to be challenged. Especially given adolescents view them as normal forms of social interaction and not as cyberbullying (54). Future research should explore ways to challenge the normalization of cyberaggression on SM.

### 4.4 Blame and responsibility

Across the themes there was an undercurrent of victim blaming, conceptualized as victims being seen as somewhat responsible for their victimization (116). Participants placed significant responsibility on individuals to use SM in ways that minimizes risk (e.g., avoiding features that allow anonymity, limiting opinion sharing, and restricting audience size), determine whether an incident constitutes cyberbullying, and take appropriate action (e.g., telling the perpetrator to stop, blocking, reporting, or deleting them). Those who failed to act in line with these expectations were negatively judged, even though a lack of action following victimization conflicted with participants’ own views that such actions were often ineffective, can escalate the situation and may then extend to the school environment. There has been minimal exploration of victim blaming in cyberbullying research, although the findings are consistent with broader cyber-based research where victim blaming was observed in studies of online fraud (117) and revenge porn (118). Similarly with the participants’ views that cyberaggression should be tolerated when seen as a predictable outcome of actions on SM, previous research showed that cyberbullying was deemed as acceptable when victims were viewed as oversharing personal information online (119, 120), highlighting a tendency to justify harmful behaviors based on targets perceived responsibility. Such attitudes can have considerable implications, as bystanders are less likely to intervene or provide support when blame was attributed towards the victim (121). Given that social support can act as a protective factor against adverse psychological outcomes linked to cyberbullying (122), addressing these tendencies is critical. Moreover, these inclinations are not confined to peers as teachers have also been found to blame adolescent victims, despite receiving training on recognizing and managing cyberbullying (105). Suggesting that cultures of blame and responsibility may be embedded at both individual and institutional levels. As discussions rarely focused on the perpetrator’s actions, it is important to consider whether prevention efforts have overemphasized the individual responsibilities of SM users. Implementing strategies that instead promote a shared social responsibility could be more effective in reducing perpetration (123). Although research on blame and responsibility in cyberbullying and cyberaggression is limited, this study provides clear evidence that adolescents’ perceptions are grounded in victim blaming narratives. Therefore, educational resources, awareness campaigns and preventative strategies should be reviewed to ensure that these narratives are not promoted, and instead reframe responsibility as a shared social obligation, rather than an individual burden.

### 4.5 Implications

The findings of this study suggest that adopting cyberaggression as a broader construct has several implications for research, education, and practice. From a measurement perspective, focusing on cyberaggression may enable more accurate and inclusive prevalence estimates by capturing a broader range of harmful online behaviors experienced by adolescents, without being impacted by the inconsistent conceptualization of the cyberbullying definition and criteria. As at present, existing measurement tools mirror these conceptual ambiguities, contributing to variability in reported prevalence rates (45, 47, 50). As digital environments continuously evolve and diversify, maintaining a coherent and valid definition of cyberbullying remains a challenge (46). Adopting the broader construct of cyberaggression may therefore offer a more flexible and inclusive approach, reducing the need to continually revise and update cyberbullying definitions and measurement tools in response to emerging forms of online aggression. In terms of intervention efforts, adopting cyberaggression would recognize that harmful online behaviors also warrant attention and support even when they do not meet traditional bullying criteria. This is particularly relevant given that cyberbullying detection models and intervention tools are often trained around the rigid and subjective cyberbullying criteria, potentially overlooking experiences that adolescents perceive as harmful (124, 125). Regarding school policy and educational initiatives, using cyberaggression may offer language that more closely aligns with the online experiences of adolescents and help to avoid misrepresenting the perspectives of young people in ways that could undermine intervention and prevention efforts (94).

In terms of clinical/psychiatric implications, adopting cyberaggression also has important consequences for clinical practice. Given the conceptual confusion and overlap between cyberbullying and cyberaggression, when an adolescent discloses either experience, clinicians should assess for mental health difficulties commonly associated with technology-facilitated abuse, such as anxiety, depression, and suicidal ideation (126). In addition, psychoeducation delivered by clinicians working with adolescents should be updated to reflect current understandings of how both cyberbullying and cyberaggression are linked to poor mental health outcomes, particularly in light of participants’ perceptions that school-based education is often outdated and unrelatable (127). Clearer guidance around breaching confidentiality may be warranted, especially in cases where perpetrators continue to engage in technology-facilitated abuse with limited regulation or accountability from SM platforms, in an attempt to prevent further harm to targeted adolescents (128). Finally, consistent with the study’s broader findings around blame and responsibility, it is important that self-blame – a hallmark of victim-blaming – be explored further to examine whether similar patterns of increased internalizing and externalizing difficulties observed in traditional bullying are also present in cyberbullying and cyberaggression (129).

### 4.6 Limitations and future research

Whilst this study has produced valuable insights into adolescents’ perceptions of cyberbullying and cyberaggression within this specific school setting, it is not without its limitations. Although the focus groups were conducted in December 2019 – January 2020, the findings remain informative despite subsequent changes in SM functionalities and safety features. Social media platforms evolve rapidly, and some behaviors described by participants may have shifted in form; however, it is likely that these will continually develop alongside the implementation and change of platform features. The behaviors used to inform adolescents’ conceptualizations of cyberaggression are also likely to evolve, further supporting the need for research to adopt cyberaggression as a more inclusive approach to studying online aggressive behaviors. Participants also highlighted how educational resources and guidance was outdated, which will remain a consistent challenge as SM platforms continue to introduce new functionalities. Therefore, the emphasis on involving adolescents directly in shaping educational resources continues to be relevant. SM platforms have also expanded their safety features. For example, Meta introduced a strike system designed to hold users accountable for repeatedly violating their community standards, by restricting access to features (130). Additional changes are anticipated through the implementation of the Online Safety Act 2023 (131), which requires SM companies to strengthen protections for young people and provide clear and accessible reporting mechanisms. Though platform reporting interfaces are still argued to contain structural barriers that discourage reporting (108). Together, this suggests that although the SM environment has evolved, the broader issues identified in this study continue to be highly relevant.

Despite the best efforts of the researcher to recruit a diverse sample, the sample was still predominantly female. Future research should look to incorporate and prioritize the voices of males and other gender identities. There is a clear need for further qualitative research and the involvement of young people more generally in research, to capture important insights within the field. Adopting a participatory action research (PAR) approach (132), where young people work alongside academic researchers to explore key topics, could allow for research in the field to be more impactful. For example, O’Brien and Doyle’s, (133) PAR approach to determine core bullying issues within a UK secondary school helped to produce important insights. As a result, the authors emphasized the value of PAR to incorporate the knowledge and perspectives of co-researchers in targeting bullying-related issues. Therefore, it could be beneficial to adopt a PAR approach to determine specific support needs regarding cyber-based aggressions in young people, including awareness, education, and support for victimization in schools and clinical practice.

### 4.7 Conclusion

This research emphasizes the importance of engaging with adolescents to understand whether their conceptualizations of cyberbullying or cyberaggression align with academic, theoretically-driven definitions. The finding that adolescents did not define cyberbullying based on all core bullying criteria, combined with persistent definitional issues and inconsistencies, suggest that focusing research efforts on cyberaggression instead may offer a more productive trajectory for the field. These perceptions were framed within the SM context, where aggression was normalized and enabled by platform features. Additionally, adolescents’ views were strongly shaped by victim-blaming narratives, placing responsibility on individuals to prevent and manage incidents and underscore the need for these beliefs to be challenged. Dissatisfaction with reporting mechanisms and outdated education highlight the importance of co-designing policies and educational resources with adolescents, and also reflect the complexities of the school environment, to ensure they are relatable and effective. Collectively, these findings highlight the importance of incorporating adolescents’ perceptions more widely in research and educational initiatives.

## Data Availability

The datasets presented in this article are not readily available because of ethical and privacy-related concerns due to the level of detail in the transcripts. Requests to access the datasets should be directed to Dr Emily Smith, emily.smith2@dmu.ac.uk.
