# Supplementary material for: A thematic analysis of adolescents’ perceptions of cyberbullying and cyberaggression: definitional distinctions and educational gaps
Source: Front Psychiatry. 2026 Jun 29;17:1746672. doi: 10.3389/fpsyt.2026.1746672 (PMC13357415; doi:10.3389/fpsyt.2026.1746672)
Supplement: Supplementary file 1 [file DataSheet1.docx]

Supplementary Material

# Supplementary Data

## Expanded Extracts

### Unhelpful and unrelatable education

*Extract 1, FG2*

| A12:  A11:  A10:  A11:  A10:  A12:  A13:  A12:  A13:  A11:  A11:  A13:  Interviewer:  A09:  A12:  A11:  A10:  A11:  A13:  A10:  A11:  A12:  A10:  A09:  A12:  A09:  A11:  A10:  A11: | The videos always like cyberstalking though  Yeah and weird stuff  It’s like they just put like you message the girl they put the like sim card or whatever it is under the grass or whatever or under the bush (.) he goes to find it puts it in his computer and uploads the photos but then like rapes her  One thing they always say but never (.) I’ve never seen happen you know where they like print picture off of people  Yeah  Like when has that ever happened  That is more cyberstalking though than cyberbullying  They never actually do cyberbullying though they’re always cyberstalking  We must have seen one in our 4 years  No they always like promote the dodgy men ones  ( )  No but like you know what I mean it’s always ones where you’ve got that woman walk in the house and they’re like ah look [yeah I know what you mean] its stupid (.) and he’s just sat in the house  Yeah I know which one  *What was you gonna say sorry?*  They just show these videos basically where (.) and that girl (.) like she walks into her house and then there’s like a sign outside with her (.) have you seen it (.) with the picture (.) and then all of a sudden the mans like there (.) like it don’t make sense  Even though she goes in first (.) it’s always really weird  And then there’s one where he like (.) have you seen the one where she’s like sat in the chippy (.) [and she’s like I can’t go home my mum will disown me] yeah that one and then the chippy lady is just like are you alright and she’s like not really (.) and there’s that one where the boys sat on the beach and that old man like put his arm out (.) it’s weird  They’re all outdated  Yeah  Yeah  They’re like (.) Blackberry’s from like 2007 [yeah] they should at least use and iPhone or like anything newer (.) to make it show how you can do it that phone instead of a little Nokia brick  I swear on one of them they’re on flip phones you know  Yeah  Exactly  Yeah like the only updated one was that one was that girl on the MacBook thing (.) on the laptop  Yeah but that one was quite old though still  Yeah but still  I think the most recent one we’ve had is when the police came in and they were like (.) oh whatever her name was and this man they were texting and then he chased her through the forest  Yeah that was the newest  I think that was the newest and that still happened what 2015 ish or something like that |
| --- | --- |

Extract 2, FG3

| A15:  A17:  A14:  A18:  A15:  A14:  A17:  A15:  A14:  A15:  A17:  A16:  A16:  A15:  A17:  A16:  A17: | Are you stupid (.) are you dumb [yes] (.) yes I am like (.) I don’t know the answer (laughs) cause people (.) people like that (.) that hurts my head (.) that could be bullying in a way like (.) the tiniest thing is bullying (.) but like it’s like (.) this school doesn’t (.) get on it like they say they have a school council who has their little sessions going on  (Laughs)  But what do they actually do? I don’t know  ( )  I don’t even know who our council members are  There’s **** and ****  I don’t even know what they do  I didn’t even know that (.) I only knew **** cause she leaves form  ( )  They get told to (.) like when I was in school council it was 5 minutes just talking about bullying but what do they actually do  They just use the kids  They can have a poster erm (.) if I’m gay I’m gay for homophobic (.) if I’m this colour I’m that colour if I’m this religion  If I’m this colour I’m that colour (laughs)  It’s true but (.) I could walk around the school and read the posters (.) but what’s the posters gonna do to me (.) make me read it (.) is it gonna make other people stop no  Improve your English skills (laughs)  ( )  It’s like they don’t think about the actual  Yeah (.) I sometimes think that a group of people should come together and do an assembly for each year to show how it can affect people and say their own  Yeah (.) we need actual people to come in school  It needs to be personal so that it gets to us  It can’t just be a teacher that’s like I went to school years ago it was easier its hard now (.) deal with it (.) it’s like |
| --- | --- |

*Extract 3, FG1*

| A03:  A04:  A03:  A04: | I don’t think you can prevent it to be honest  Yeah yeah you can’t [okay] cause like think how many millions of people are using social media and things like that (.) if one of your tweets or anything or one of your posts blows up (.) because you’ve said something (.) then people have that people will save that people will screenshotted that (.) you’ve gotta (.) you know take it or just don’t say anything like that  Like I think the best shot is to educate people more (.) but even then (.) you know you can minimise it if everyone stops using social media which is not gonna happen (0.2) it’s the same with limiting screen time (.) you can’t really do anything to do that (.) cause like kids are gonna use social media regardless of what you put in place so I guess that’s the same with like aggression maybe  And the behaviours becoming like more acceptable with like young kids because you see like a lot of (.) you know 10-year olds with phones with like all these different accounts (.) they’re now exposed to all those kind of behaviours that adults are displaying (.) and they think that’s acceptable” |
| --- | --- |

### The complexity of the school environment

*Extract 4, FG3*

| *Interviewer:*  A15:  A17:  A15:  A17:  A15:  A17:  A15:  A17:  A15:  *Interviewer:*  A15:  A17:  A15:  A17:  A14:  A17:  A15:  A17:  A15:  A17: | *Okay (.) so is there anyone that don’t think that they are serious (.) or do you think that maybe there are some behaviours that are more serious than others?*  Yeah definitely (.) I’d say if someone just calls you a (.) slag on a post like [yeah] you can just ignore it delete the comment [deal with it] so many people keep the comment (.) delete them (.) block them (.) gone  You know on people’s stories where it’s like (.) you’ve got that ask me a question thing (.) and they always keep the ones where it’s like show me a nude (.) it’s like  Or they post that one and not the one saying like (.) I don’t know ermm (.) you’re really pretty or something it has to be Peng or something like that  Are we on about ****? Are we both on about ****?  Probably (laughs) (.) like it’s like the things that go on its just like (.) what’s the point (.) like I like if it’s on my Instagram I just put funny things on there (.) I put such weird things  I don’t answer all of them because there’s no point  Some of them are just like what’s the point (.) like they’ll call you a slag on there and you’re like okay thank you  Honestly I can’t say I’ve ever had that one  I have because people don’t like me  *So they’re like the more less serious things?*  Yeah like you’ve gotta learn that those people are just losers and a waste of time  Yeah  Cause if they can just go on there (.) take their time (.) like 5 seconds of their life to type that comment (.) send it and wait for them to reply to start and argument then (.) your life’s boring  Yeah but then they complain (.) they complaint about the negative things they’ve got like (.) right either delete the negative things (.) or don’t answer it (.) or don’t do the ask me a question thing again  Or block them (.) like so many people keep them people on their Instagram’s and their Snapchat’s because they want an argument (.) it’s not the fact that [yeah that’s true] they could be crying to their friends (.) they could be crying to their mums and dad but yet (.) they’re still on your Instagram able to send these comments able to look at your (.) stories and your photos so what’s the point (.) block them  Yeah but also at the same time (.) like sometimes blocking them or unfriending them causes a bigger argument  Yeah it makes no sense because like  Especially if you’re in school (.) if you like (.) say you block someone like (.) out of school (.) they’re gonna come up to you and ask you why you’ve blocked them (.) like its (.) sometimes it’s not worth it  Or they’ll tell all your friends and then they’ll ask you  Yeah like can you ask so and so like no I did it for a reason |
| --- | --- |

*Extract 5, FG1*

| A06:  A04:  A05:  *Interviewer:*  A05:  A01:    A05:  A02:  A06:  A03:  A02:  A03:  A04:  A03:  A02:  *Interviewer:*  A04:  *Interviewer:*  A06:  A01:  *Interviewer:*  A01:  *Interviewer:*  A05: | Tell the teachers (.) there’s more teachers than students  There you go you don’t fight  If you go to the teachers they’re gonna get other people and then it will escalate even more  ( )  *So you don’t necessarily think telling the teachers is a good idea?*  No because they don’t do anything  They don’t really do anything  Not most of them do anything but it will just escalate even more because they’ll be like because you’ve told the teacher I'm gonna get told of now and then they’ll just do it even more  It depends what level they are like (.) head teacher teacher or like support  Yeah but if you tell the teacher then they’ll probably escalate it to out of school (.) and then like get you when you’re out of school  But even if you do tell a teacher they can’t physically from that voicing their opinion (.) so it’s more you just need to be sensible and like back down  You’re not gonna back down if you’re right are you?  Yeah but then it’s sensible if they won’t shut up  You just have to be mature yeah  Yeah there’s no point just getting your hands dirty (.) just to fall to a lower level  It depends how much it annoys you (.) if you like just think (.) its constant and you now it’s not true (.) not speaking the truth and then you can just leave it  *Okay so is it any kind of you know you mentioned about different levels of different teachers or like head teachers (.) I dunno if you have like teaching assistants or like class support [yeah] is there anyone you would feel comfortable (.) telling or is there?*  To be honest I’d rather tell like a T.A [yeah] an assistant than like I would [an actual teacher] yeah because I feel like they’re more (.) [understanding] yeah  *Yeah what about you? Do you guys (.) do you think anything different? Is there someone you’d feel comfortable going to or do you disagree?*  There’s like certain that would listen and certain which would [yeah] just be like oh well  And some teachers don’t like you and some teachers do (.) so it depends on who it is  *So you’d feel more comfortable going to the teachers that you think like you more than the ones that don’t?*  Yeah  *Okay yeah*  I’d just go to the ones that are nice |
| --- | --- |

*Extract 6, FG2*

| A09:  *Interviewer:*  A10:  A12:  A11:  *Interviewer:*  A12:  A11:  A09:  A13:  A09:  *Interviewer:*  A09:  A10:  *Interviewer:*  A10:  A09:  A11:  A12:  *Interviewer:*  A11:  *Interviewer:*  A09:  A11: | Or report (.) that’s what you get told to do  *Oh so you get told to block or report them?*  Yeah  Yeah but sometimes the report doesn’t even work  No  *Is that on social media or do you mean to someone?*  Yeah  On social media but if you go to a teacher in school and be like oh so and so say this online or blah blah blah is doing this (.) most of the time (.) instead of like sometimes in really serious cases they call the other person up but a lot of the time they’re just like block them or report them [yeah] so it’s just not helpful it’s just like  Or they get the police in school and you have a meeting (.) that’s what happens to most people  Yeah depending on how serious it is  Yeah  *Oh so if it gets quite bad they get the police in to talk to [yeah] the person who’s doing it?*  To both of them  To both of you together in the same room  *Oh okay (.) do you think that’s useful or?*  No  No  No  I have never been involved in it to be fair  *If that was like (.) obviously you know that’s an option if it gets that bad [yeah] [yeah] if it gets that bad like would you feel comfortable like reporting it knowing that that would be like*  No not really  *No oh so you don’t really like that kind of strategy?*  No  No it makes it a bit (.) I mean I think it serious but when the police get involved it’s a bit more (.) yeah and it’s just a bit (.) really like (.) it was bad but it wasn’t like (.) have to get the authorities in bad and I think it’s just a bit (.) erm stressful for those people cause ones obviously gonna be like oh no look what I’ve done and the other ones like oh god [yeah] (.) I’ve let this happen” |
| --- | --- |

### The crossover from cyber aggression into cyberbullying / Unclear definitional distinctions surrounding cyberbullying and cyber aggression

*Extract 7, FG2*

| *Interviewer:*  A09:  A10:  A11:  A12:  *Interviewer:*  A13:  A11:  A13  *Interviewer:*  A10:  A13:  A11:  *Interviewer:*  A11:  A10:  A13:  *Interviewer:*  A11:  A12:  *Interviewer:*  A13:  *Interviewer:*  A10: | *So kind of on the repeated thing (.) how many times then would you say is (.) does it become bullying?*  After you tell them to stop  Yeah  Yeah  Yeah  *Okay*  I think because you can have erm like running jokes with your mates where you keep like taking the mick out of them but it’s at that point when they say stop and you keep going (.) I think  Or when like the joke dies or something and they just repeatedly [keep bringing it] they don’t necessarily say stop but it’s like (.) it’s an old joke and they keep bringing it up  And it’s clear you wanted them to stop [yeah] you might not have specifically said it but (.) when its ended and they just keep going  *So how often or how many times would you have to experience that for you to tell them to stop (.) like when you believe*  When you feel insecure about what they’ve said  Yeah  Or when it’s not just like (.) mentioned in a conversation where you’re having a laugh (.) where it’s just mentioned like you’ll just be talking about homework or something and they’ll just be like alright and then use a nickname that pokes fun at you  *So a little bit unprovoked then?*  Yeah  Yeah  Yeah  *So just out of nowhere so there’s no kind of specific amount of times [no] you would say (.) do you just go by how you feel or?*  Yeah  Yeah it’s all personal as well because some people can go further and be able to deal with it more than other people  *Okay*  Yeah so it’s just whenever it hurts you  *Yeah so then do you think then different people have different kind of*  I think everyone has a different stopping point (.) so some person would just like say it once and be like please stop and someone could be like take it ten times and they want it to stop or they could just keep on taking it until they couldn’t take it anymore” |
| --- | --- |

*Extract 8, FG3*

| *Interviewer:*  A15:  *Interviewer:*  A15:  *Interviewer:*  A15:  A16:  A15:  A14:  A15:  A17:  A18:  *Interviewer:*  A16:  A15:  A16:  A15: | *Okay so we’ve spoken about a lot of different behaviours but (.) how would you if I was to ask you to define online aggression (.) so like (.) how would you define if it like*  Like the definition of it?  *Yeah*  Like ermm  *Like if you could provide a definition of like (.) negative online aggressive*  Making someone feel (.) unworthy  Degrading someone  Yeah  Yeah  Like (.) tryna make someone (.) oh its hard isn’t it when you actually think about it  Belittling someone  Yeah  *Okay*  Trying to make them feel bad  Making someone feel small (.) just from words you say  So they don’t tell other people what you’ve been telling them  Yeah |
| --- | --- |

*Extract 9, FG1*

| *Interviewer:*  A03:  *Interviewer:*  A03:  A04:  *Interviewer:*  A05:  *Interviewer:*  A02:  *Interviewer:*  A02:  *Interviewer:*  A04: | *Okay so do you think then there is a difference between like the behaviours we first spoke about and online bullying? (0.4)*  Erm I think (.) bullying more penalising someone for themselves and then like aggression is more for their opinion maybe or sometimes they can overlap (.) but if you were to separate them then yeah  *So do you think then the you know the behaviours we first spoken about you know the acts of online aggression do you think they’re different to online bullying or do you think they’re the same or?*  I think one leads to the other  Yeah (.) it’s quite a fine line but (.) it yeah like online aggression just turns into online bullying eventually  *Okay what do you girls think?*  That (.) yeah like (.) either way online aggression you’re still criticising someone or something that they believe in or think that is right to them (.) and then you just carry on and carry on and then it just like becomes bullying and then like someone can’t take it anymore and they might just kill themselves  *Yeah what do you think about it? Do you think they’re the same different?*  It like depends on the level like (.) because aggression can be to a higher level or a lower level and bullying can be on a higher level and a lower level (.) but if they’re both on a higher level then it’s actually quite serious (.) if they’re both on a higher level  *Do you think then they’re similar the same different or?*  They’re quite similar because you’re still attacking someone (.) because of their thoughts or they’re views or because of the way they look  *Okay yeah so is there anything else?*  Probably just yeah (.) online aggression is just online bullying but (.) once online bullying is online aggression just several times |
| --- | --- |

### Social media as a tool for perpetration

*Extract 10, FG2*

| *Interviewer:*  A12:  A11:  A12:  A11:  *Interviewer:*  A12:  *Interviewer:*  A10:  *Interviewer:*  A11:  A09:  A10:  A09:  A12:  *Interviewer:*  A09:  A10:  A12:  A11:  A10:  *Interviewer:*  A11:  A13:  A12:  A11:  A13:  A12: | *Sorry (.) erm okay so is there anything else then that you spot that happens online? (0.4)*  There are a lot of negative things like especially (.) on peoples (.) anonymous things you get a lot of (.) bullying for like LGBT reasons  Yeah and there’s just like (.) a lot of people just because they’re anonymous saying a lot of things  Because they don’t have the face to actually say it  Yeah  *Oh okay so this is on snapchat?*  Yeah  *And you don’t necessarily know who the person is?*  No cause you can do this thing and then you can send an anonymous message  *Oh can you?*  Yeah a lot of people send like nice things  Yeah you put like ask questions  And then they just send stuff  Yeah or they just put like negative comments  Yeah some of them are nice and then obviously some just go a bit far  *Is that a little bit like the thing on Instagram where you ask the questions*  Yeah  Yeah  Yeah  Yeah  It’s like that but you just can’t see the people who it is  (...)  *Okay erm (.) so how do you think then some of like the aggressive behaviours that you’ve spoken about (.) how do you think they make people feel?*  Upset  Yeah definitely  I mean you get a lot of jokingly aggressive behaviours [oh yeah] between friends but then it’s the actual (.) like the other side of it that like  I think on the anonymous websites the fact that they don’t know who is saying it can make them feel really insecure (.) because if it’s like you know who’s saying it to you (.) you can just put it down as oh they don’t like me but if you don’t know it’s just a bit like well who thinks that  Yeah it can be overwhelming  Especially if you get a lot of them |
| --- | --- |

*Extract 11, FG3*

| A17:  A15:  *Interviewer:*  A15:  *Interviewer:*  A15:  *Interviewer:*  A14:  A17:  A15:  *Interviewer:*  A17:  A15:  A17:  A15:  *Interviewer:*  A14:  A15:  A17: | No but Snapchat was like made to (.) [yeah photos] sent nude photos (laughs)  Except for the subscribe bit that usually has things like fights on it [yeah] sometimes  *Okay*  Snapchats more about people’s stories then actual videos you can see  So do people post like you were saying how someone has an argument they’ll post that on their Instagram (.) do they do that on Snapchat as well or?  Yeah they do it on any social media site  *Oh really?*  It’s like a thing  And private stories (0.2) private stories have a lot of gossip  Private stories are the worst because you put people on your private (.) that they shouldn’t be the people on the private story because they’ll then go and tell the person (.) like  *So is that like the close friend’s bit where you can just send it to certain people?*  Yeah  Yeah it’s on Snapchat as well but you can have [oh okay] like as  Snapchat you can have like loads  Close friends you can have like thirty but Snapchat you have like (.) 300 or as many as you want to be honest  *Oh okay (.) so people send like private gossip or something?*  Yeah  Sometimes or they’ll just put like where they are and what they’re doing but they don’t [yeah] they don’t want all their followers to see (.) just certain people  Yeah |
| --- | --- |

*Extract 12, FG1*

| *Interviewer:*  A02:  *Interviewer:*  A02:  *Interviewer:*  A06:  A01:  *Interviewer:*  A01:  A06:  A04:  A06:  A04: | *Okay yeah (.) erm so what would you guys do if you ever was on the receiving end of and of these behaviours? (0.3)*  Probably report it and then block them (.) keep the evidence  *Okay so how would you report it?*  Probably at the report button  *Oh so like on the social media account you’d report it that way*  Sometimes that doesn’t work though  Block um  *The reporting?*  Yeah because they make new accounts and then harass you  And put your account on private  I feel like (0.3) a lot of people (.) it’s not very like proactive (.) a lot of people say (.) I am gonna block them and keep the evidence and report it or whatever (.) and it just doesn’t happen (.) I don’t think I’ve ever seen someone actually like report someone because they’re being attacked or whatever  My cousin does it all the time  On social media |
| --- | --- |

# Supplementary Figures and Tables

**Table 1** *Participant demographics by focus group.*

| Participant ID | Age | Ethnicity | Gender | Social Media Accounts |
| --- | --- | --- | --- | --- |
| Focus Group 1 | | | | |
| A01 | 14 | English/Welsh/Scottish/Northern Irish/British | Female | Facebook, Instagram, Snapchat, YouTube, Messenger (Facebook), WhatsApp, TikTok & Skype |
| A02 | 14 | Indian | Male | YouTube & Whatsapp |
| A03 | 15 | Indian | Female | YouTube & Whatsapp |
| A04 | 15 | English/Welsh/Scottish/Northern Irish/British | Male | Twitter, Instagram, Snapchat, YouTube & WhatsApp |
| A05 | 14 | English/Welsh/Scottish/Northern Irish/British | Female | Instagram, Snapchat, YouTube, Messenger (Facebook), WhatsApp & TikTok |
| A06 | 14 | English/Welsh/Scottish/Northern Irish/British | Female | Instagram, Snapchat, YouTube, Messenger (Facebook), WhatsApp & TikTok |
| Focus Group 2 | | | | |
| A07 | 14 | English/Welsh/Scottish/Northern Irish/British | Female | Snapchat, Messenger (Facebook) & TikTok |
| A08 | 14 | English/Welsh/Scottish/Northern Irish/British | Female | Facebook, Instagram, Snapchat, YouTube, Messenger (Facebook) & WhatsApp |
| A09 | 15 | English/Welsh/Scottish/Northern Irish/British | Female | Facebook, Instagram, Snapchat, Messenger (Facebook) & TikTok |
| A10 | 14 | White & Black Caribbean | Female | WhatsApp & TikTok |
| A11 | 14 | English/Welsh/Scottish/Northern Irish/British | Female | Twitter, Instagram, Snapchat, YouTube, WhatsApp & TikTok |
| A12 | 14 | English/Welsh/Scottish/Northern Irish/British | Female | Twitter, Instagram, Snapchat, YouTube, WhatsApp & TikTok |
| A13 | 14 | White & Asian | Female | Twitter, Instagram, Snapchat, YouTube & WhatsApp |
| Focus Group 3 | | | | |
| A14 | 14 | African | Female | Instagram, Snapchat, YouTube, WhatsApp & TikTok |
| A15 | 15 | English/Welsh/Scottish/Northern Irish/ British | Female | Instagram, Snapchat, YouTube, WhatsApp & TikTok |
| A16 | 15 | White European | Female | Facebook, Instagram, Snapchat, YouTube, WhatsApp & TikTok |
| A17 | 15 | English/Welsh/Scottish/Northern Irish/ British | Female | Instagram, Snapchat & YouTube |
| A18 | 14 | English/Welsh/Scottish/Northern Irish/ British | Female | Facebook, Instagram, YouTube, Messenger (Facebook) & WhatsApp |
